# Residential social vulnerability among healthcare personnel with and without severe acute respiratory coronavirus virus 2 (SARS-CoV-2) infection in Five US states, May–December 2020

**DOI:** 10.1017/ice.2023.131

**Published:** 2024-01

**Authors:** Maria Zlotorzynska, Nora Chea, Taniece Eure, Rebecca Alkis Ramirez, Gregory T. Blazek, Christopher A. Czaja, Helen Johnston, Devra Barter, Melissa Kellogg, Catherine Emanuel, Ruth Lynfield, Ashley Fell, Sarah Lim, Sara Lovett, Erin C. Phipps, Sarah Shrum Davis, Marla Sievers, Ghinwa Dumyati, Cathleen Concannon, Christopher Myers, Kathryn McCullough, Amy Woods, Christine Hurley, Erin Licherdell, Rebecca Pierce, Valerie L.S. Ocampo, Eric Hall, Shelley S. Magill, Cheri T. Grigg

**Affiliations:** 1 Division of Healthcare Quality Promotion, National Center for Emerging and Zoonotic Infectious Diseases, Centers for Disease Control and Prevention, Atlanta, Georgia; 2 Chenega Enterprise Systems & Solutions, LLC, Chesapeake, Virginia; 3 Colorado Department of Public Health and Environment, Denver, Colorado; 4 Minnesota Department of Health, St. Paul, Minnestoa; 5 New Mexico Emerging Infections Program, University of New Mexico, Albuquerque, New Mexico; 6 New Mexico Department of Health, Santa Fe, New Mexico; 7 New York Emerging Infections Program, University of Rochester Medical Center, Rochester, New York; 8 Public Health Division, Oregon Health Authority, Portland, Oregon; 9 School of Public Health, Oregon Health and Science University, Portland, Oregon

## Abstract

**Objective:**

To characterize residential social vulnerability among healthcare personnel (HCP) and evaluate its association with severe acute respiratory coronavirus virus 2 (SARS-CoV-2) infection.

**Design:**

Case–control study.

**Setting:**

This study analyzed data collected in May–December 2020 through sentinel and population-based surveillance in healthcare facilities in Colorado, Minnesota, New Mexico, New York, and Oregon.

**Participants:**

Data from 2,168 HCP (1,571 cases and 597 controls from the same facilities) were analyzed.

**Methods:**

HCP residential addresses were linked to the social vulnerability index (SVI) at the census tract level, which represents a ranking of community vulnerability to emergencies based on 15 US Census variables. The primary outcome was SARS-CoV-2 infection, confirmed by positive antigen or real-time reverse-transcriptase– polymerase chain reaction (RT-PCR) test on nasopharyngeal swab. Significant differences by SVI in participant characteristics were assessed using the Fisher exact test. Adjusted odds ratios (aOR) with 95% confidence intervals (CIs) for associations between case status and SVI, controlling for HCP role and patient care activities, were estimated using logistic regression.

**Results:**

Significantly higher proportions of certified nursing assistants (48.0%) and medical assistants (44.1%) resided in high SVI census tracts, compared to registered nurses (15.9%) and physicians (11.6%). HCP cases were more likely than controls to live in high SVI census tracts (aOR, 1.76; 95% CI, 1.37–2.26).

**Conclusions:**

These findings suggest that residing in more socially vulnerable census tracts may be associated with SARS-CoV-2 infection risk among HCP and that residential vulnerability differs by HCP role. Efforts to safeguard the US healthcare workforce and advance health equity should address the social determinants that drive racial, ethnic, and socioeconomic health disparities.

Healthcare personnel (HCP) were among the first known cases of severe acute respiratory coronavirus virus 2 (SARS-CoV-2) infection and represented one of the highest risk groups for acquiring the virus during the height of the coronavirus disease 2019 (COVID-19) pandemic.^1,2^ Although HCP who care for patients with COVID-19 experience infection risk within their occupational settings, community exposures also represent a substantial source of transmission among HCP.^3,4^ Much like in the general population, racial and ethnic disparities in infection rates, specifically among Black and Hispanic HCP, have been widely documented in the literature.^5–8^ However, the mechanisms driving these disparities among HCP are not well understood.

Epidemiologists have long recognized the impact of geospatial determinants on infectious disease transmission. Poverty, crowded housing, and other markers of socioeconomic disadvantage increase community vulnerability to adverse outcomes during pandemics. Furthermore, racial residential segregation persists in the United States; predominantly Black and Hispanic neighborhoods experience high levels of economic disinvestment and marginalization.^9^ In their conceptual framework for how health disparities arise during an influenza pandemic, Blumenshine et al^10^ identified differential exposure as one of the primary drivers. Housing density, increased reliance on public transportation, and inability to remain at home for those working in lower-wage occupations are among the ways socioeconomic factors increase exposure risk. Barriers to healthcare access, including testing and treatment, further exacerbate these disparities.^11–13^ This framework can be applied to other respiratory viral pandemics like COVID-19. Area-level studies have found that neighborhoods and counties with higher proportions of racial and ethnic minority groups and those of lower socioeconomic status experienced higher rates of SARS-CoV-2 cases, mortality, and morbidity.^14^ Therefore, operationalizing and quantifying exposure to residential social vulnerability can further understanding of this social determinant of health within high-risk populations in the COVID-19 pandemic, such as HCP.

The extent to which social and economic factors in the residential environment influence SARS-CoV-2 acquisition risk among HCP remains unknown. Elucidating these relationships may help guide interventions that safeguard the US healthcare workforce and further health equity among HCP. In this case–control analysis, we used data collected in collaboration with the CDC Emerging Infections Program^15^ to characterize residential social vulnerability and its association with SARS-CoV-2 acquisition among HCP working in select healthcare facilities in 2020.

## Methods

### Participant enrollment and data collection

The methods for data collection have been previously described.^4^ Staff at EIP sites in 5 states (Colorado, Minnesota, New Mexico, New York, and Oregon) recruited personnel from a convenience sample of healthcare facilities and healthcare systems. EIP staff obtained weekly lists of HCP tested for SARS-CoV-2 via reverse-transcription polymerase chain reaction (RT-PCR) or antigen test (hereafter both referred to as virus test) from participating healthcare facilities, or state or local health departments through routine disease surveillance. Protocols for testing varied by facility, with some conducting routine screening programs and others testing HCP who presented with symptoms or suspected exposure. EIP staff attempted to contact all HCP with positive virus test results and enroll all who agreed to participate. EIP staff randomly selected HCP with negative virus test results from the same week and facility as a positive case to contact and enroll in the study. A larger proportion of controls did not respond to contact attempts or declined participation in the study as compared to cases.^4^ Therefore, controls were selected via incidence density sampling.^16^ All HCP were interviewed within 60 days of a positive or negative test.

EIP staff conducted telephone interviews of consenting HCP using a standardized questionnaire. This interview included questions about basic demographics (ie, residential address, sex, age, race, and ethnicity), HCP job role, SARS-CoV-2 exposures both within and outside the workplace, and detailed COVID-19 patient care activities in the 14 days before specimen collection (asymptomatic HCP) or COVID-19 symptom onset (symptomatic HCP). Project data were collected and managed using REDCap (Research Electronic Data Capture), a secure, web-based software platform designed to support data capture for research studies.^17,18^ REDCap provides an intuitive interface for validated data capture, audit trails for tracking data manipulation and export procedures, automated export procedures for seamless data downloads to common statistical packages, and procedures for data integration and interoperability with external sources.

This activity was reviewed by the and was conducted in compliance with applicable federal law and CDC policy (45 CFR part 46.102(l)(2); 21 CFR part 56; 42 USC §241(d); 5 USC §552a; 44 USC §3501 et seq). The CDC determined that the project was a non-research activity, and no institutional review board review was required. EIP sites and participating facilities either deemed the project to be a nonresearch activity or obtained institutional review board approval.

### Case and control definitions

Cases were defined as HCP working in participating healthcare facilities who had a positive SARS-CoV-2 virus test from samples collected between May 19, 2020, and December 31, 2020. Controls were defined as HCP who had a negative SARS-CoV-2 virus test during the same period and worked in the same healthcare facilities as cases. Controls were eligible to participate multiple times provided they did not have a previous positive SARS CoV-2 test. HCP who had previously tested positive were not eligible to participate as controls.

### Exposures and covariates

The primary exposure of interest was the 2020 Social Vulnerability Index (SVI) for the census tract where the HCP resided. The Agency for Toxic Substances and Disease Registry (ATSDR) created this composite measure to identify communities most in need of support before, during, and after hazardous events, such as infectious disease outbreaks.^19^ The SVI comprises 15 US Census variables, grouped within 4 themes: socioeconomic status, household characteristics, racial and ethnic minority status, and housing type and transportation. The overall SVI and each of the 4 themes are continuous variables from 0 to 1, representing the percentile rank among all US Census tracts with higher values representing higher social vulnerability.^20^

EIP staff geocoded residential addresses to census tracts using 2020 US Census shapefiles. We then merged geocoded participant data with a dataset of SVI values at the census-tract level. We categorized the overall SVI as a dichotomous variable, with the highest quartile of the sample representing “high SVI” and the lower 3quartiles representing “low SVI.” We created similar dichotomous variables for each of the SVI themes.

Other participant characteristics included EIP site, type of facility where HCP worked at the time of virus test (eg, acute-care hospital, nursing home, outpatient clinic, other), age (<30 years, ≥30 years), race and ethnicity, and healthcare role [registered nurse; administrative personnel; certified nursing assistant (CNA); physician; medical assistant; and other, based on anticipated levels of patient contact as determined by the researcher’s assessment of the HCP role as substantial, moderate, minimal, or undefined). We also assessed whether HCP had close contact with COVID-19 patients in the workplace and whether HCP assisted COVID-19 patients with their activities of daily living (ADL; eg, bathing, eating, toileting). These workplace exposures were assessed for the 14 days before symptom onset or virus test specimen collection date (if asymptomatic).

### Statistical analysis

The analytic sample was restricted to all cases and controls who provided residential addresses that could be geocoded to valid US census tracts. Furthermore, only observations from facilities that enrolled both cases and controls were included in the analysis.

Descriptive statistics for participant characteristics were calculated for cases and controls. Participant characteristics by case and control status, and by SVI were compared using the Fisher exact test.

Logistic regression modeling was used to calculate the odds ratio (OR) and 95% confidence interval (CI) for the association between high SVI and case status, as well as high SVI and each of the following covariates: facility type, healthcare role, close contact with COVID-19 patients, and assisting COVID-19 patients with their ADL. We selected this initial set of variables for potential inclusion in the final multivariable model based on a directed acyclic graph (DAG) as well as our previous research using this dataset, in which we found significant associations between these variables and case status.^4^ Backwards selection at α = 0.05 was used to determine the final set of covariates included in the multivariable model, and adjusted odds ratios (aORs) and 95% CIs were calculated for associations with high SVI.

A logistic regression model that included all 4 SVI themes, as well as covariates, was used to estimate the associations between SVI themes and case status. All analyses were conducted using SAS version 9.4 statistical software (SAS Institute, Cary, NC).

## Results

In total, 2,168 HCP were included in the analysis, and 194 observations were excluded due to incomplete residential address data. The characteristics of HCP cases (n = 1,571) and controls (n = 597) are presented in Table 1. A greater proportion of cases compared to controls (28.1% vs 16.9%; *P* < .0001) lived in a census tract with an SVI in the highest quartile of the sample. Across the 4 SVI themes, a higher proportion of cases than controls resided in upper-quartile census tracts for all themes except the housing type and transportation theme. Most cases and controls worked in hospitals. Higher proportions of cases than controls identified as being Hispanic/Latino, non-Hispanic Black, age <30 years, working as administrative personnel, CNA or medical assistant, or assisting COVID-19 patients with their ADL. Approximately one-third of cases and controls reported close contact with COVID-19 patients in the workplace in the 14 days before illness onset or SARS-CoV-2 virus test-specimen collection.

**Table 1.** Characteristics of Healthcare Personnel With (Cases) and Without (Controls) SARS-CoV-2 Infection, 5 US Emerging Infections Program Sites, May–December 2020

Characteristic	Cases (n=1,571), No. (%)	Controls (n=597), No. (%)	*P* Value^a^
**SVI**^**b**^ **, highest quartile**			
Overall SVI	441 (28.1)	101 (16.9)	<.0001
Socioeconomic status	440 (28.0)	102 (17.1)	<.0001
Household composition and disability	432 (27.5)	110 (18.4)	<.0001
Minority status and language	416 (26.5)	126 (21.1)	.01
Housing type and transportation	401 (25.5)	140 (23.5)	.35
**Site location**			<.0001
Colorado	431 (27.4)	163 (27.3)	
Minnesota	204 (13.0)	130 (21.8)	
New Mexico	136 (8.7)	81 (13.6)	
New York	422 (26.9)	98 (16.4)	
Oregon	378 (24.1)	125 (20.9)	
**Facility type**			.007
Acute-care hospital	1137 (72.4)	415 (69.5)	
Outpatient clinic	231 (14.7)	115 (19.3)	
Nursing home	124 (7.9)	30 (5.0)	
Other facility type^c^	79 (5.0)	37 (6.2)	
**Age**			.002
<30 y	466 (29.7)	133 (22.3)	
≥30 y	1094 (69.6)	459 (76.9)	
Not reported	11 (0.7)	5 (0.8)	
**Sex**			.23
Female	1213 (77.2)	468 (78.4)	
Male	349 (22.2)	122 (20.4)	
Other or not reported	9 (0.6)	7 (1.2)	
**Race and ethnicity**			<.0001
White, non-Hispanic	925 (58.9)	410 (68.7)	
Hispanic or Latino, any race or races	347 (22.1)	92 (15.4)	
Black, non-Hispanic	169 (10.8)	27 (4.5)	
Asian, non-Hispanic	66 (4.2)	30 (5.0)	
Other or multiple races, non-Hispanic or race or ethnicity not reported	64 (4.1)	38 (6.4)	
**Healthcare role**			<.0001
Registered nurse	497 (31.6)	202 (33.8)	
Administrative personnel	179 (11.4)	52 (8.7)	
Certified nursing assistant	143 (9.1)	28 (4.7)	
Physician	82 (5.2)	56 (9.4)	
Medical assistant	71 (4.5)	22 (3.7)	
Other role anticipated to have substantial patient contact^d^	255 (16.2)	112 (18.8)	
Other role anticipated to have moderate patient contact^e^	202 (12.9)	67 (11.2)	
Other role anticipated to have minimal patient contact^f^	109 (6.9)	30 (5.0)	
Other role with undefined patient contact	33 (2.1)	28 (4.7)	
**Close contact with patients with COVID-19 in the workplace in the 14 d before illness onset or SARS-CoV-2 test specimen collection date**			.11
Yes	593 (37.8)	203 (34.0)	
No	978 (62.3)	394 (66.0)	
**Assisted COVID-19 patients with activities of daily living**			.0002
Yes	402 (25.6)	108 (18.1)	
No	1169 (74.4)	489 (81.9)	

Note. COVID-19, coronavirus disease; SARS-CoV-2, severe acute respiratory syndrome coronavirus 2; SVI, social vulnerability index.

a Fisher exact test.

b 2020 Social Vulnerability Index for the census tract where the HCP resided.

c Includes pharmacies, urgent care clinics, free-standing emergency rooms or departments, and mental health facilities.

d Includes dental healthcare provider, emergency medical services personnel, licensed practical nurse, nurse practitioner, occupational therapist, other nurse, physician assistant, physical therapist or assistant, phlebotomist, respiratory therapist, radiology technician, speech-language pathologist, and surgical, medical, or emergency technician.

e Includes nonphysician behavioral health provider, chaplain, care coordinator, dietician, environmental services personnel, food services personnel, patient transport personnel, research personnel, social worker, or student.

f Includes facilities maintenance personnel, medical equipment technician, laboratory personnel, or pharmacist.

Higher proportions of non-Hispanic Black (45.9%) and Hispanic (44.0%) HCP, as well as CNAs (48.0%) and medical assistants (44.1%) lived in the most vulnerable tracts compared to other racial and ethnic groups and HCP roles (Table 2). There were no statistically significant differences in the proportions of HCP living in the highest SVI quartile tracts by sex (Table 2).

**Table 2.** Census Tract Social Vulnerability Index (SVI) by Participant Characteristics Among Healthcare Personnel, 5 US Emerging Infections Program sites, May–December 2020

Characteristic	Total	High SVI, No. (%)^a^	Low SVI, No. (%)^b^	*P* Value^c^
**Sex**				.09
Female	1,681	407 (24.2)	1,274 (75.8)	
Male	471	128 (27.2)	343 (72.8)	
Other or not reported	16	7 (43.8)	9 (56.3)	
**Race and ethnicity**				<.0001
White, non-Hispanic	1,335	207 (15.5)	1,128 (84.5)	
Hispanic or Latino, any race or races	439	193 (44.0)	246 (56.0)	
Black, non-Hispanic	196	90 (45.9)	106 (54.1)	
Asian, non-Hispanic	96	21 (21.9)	75 (78.1)	
Other or multiple races, non-Hispanic or race or ethnicity not reported	102	31 (30.4)	71 (69.6)	
**Facility type**				.03
Acute-care hospital	1,552	374 (24.1)	1,178 (75.9)	
Outpatient clinic	154	88 (25.4)	258 (74.6)	
Nursing home	290	54 (35.1)	100 (64.9)	
Other facility type^d^	172	26 (22.4)	90 (77.6)	
**Healthcare role**				<.0001
Registered nurse	699	111 (15.9)	588 (84.1)	
Administrative personnel	231	67 (29.0)	164 (71.0)	
Certified nursing assistant	171	82 (48.0)	89 (52.0)	
Physician	138	16 (11.6)	122 (88.4)	
Medical assistant	93	41 (44.1)	52 (55.9)	
Other role anticipated to have substantial patient contact^e^	367	70 (19.1)	297 (80.9)	
Other role anticipated to have moderate patient contact^f^	269	93 (34.6)	176 (65.4)	
Other role anticipated to have minimal patient contact^g^	139	44 (31.7)	95 (68.3)	
Other role with undefined patient contact	61	18 (29.5)	43 (70.5)	

Note. COVID-19, coronavirus disease; SVI, social vulnerability index.

a Highest quartile of SVI values for census tracts where HCP resided.

b Lowest 3 quartiles of SVI values for census tracts where HCP resided.

c Fisher exact test.

d Includes pharmacies, urgent-care clinics, free-standing emergency rooms or departments, and mental health facilities.

e Includes dental healthcare provider, emergency medical services personnel, licensed practical nurse, nurse practitioner, occupational therapist, other nurse, physician assistant, physical therapist or assistant, phlebotomist, respiratory therapist, radiology technician, speech-language pathologist, and surgical, medical, or emergency technician.

f Includes nonphysician behavioral health provider, chaplain, care coordinator, dietician, environmental services personnel, food services personnel, patient transport personnel, research personnel, social worker, or student.

g Includes facilities maintenance personnel, medical equipment technician, laboratory personnel, or pharmacist.

In an unadjusted logistic regression model, cases were significantly more likely than controls to reside in the high SVI census tracts (odds ratio [OR], 1.92; 95% confidence interval [CI], 1.51–2.44) (Table 3). This association remained significant in the final adjusted model, which included HCP role and assisting COVID-19 patients with their ADL (aOR, 1.76; 95% CI, 1.37–2.26). In a model in which each of the 4 SVI themes was treated as an independent exposure, adjusting for HCP role and assisting COVID-19 patients with their ADL, only the socioeconomic status (aOR, 1.77; 95% CI, 1.30–2.39) and household composition and disability (aOR, 1.36; 95% CI, 1.05–1.78) themes had significant associations with case status (Table 4).

**Table 3.** Multivariable Logistic Regression Model Estimating Associations With SARS-CoV-2 Infection Among Healthcare Personnel, 5 US Emerging Infections Program Sites, May–December 2020

Characteristic	OR (95% CI)	aOR (95% CI)
**SVI**		
High^a^	1.92 (1.51–2.44)	1.76 (1.37–2.26)
Low^b^	Referent	Referent
**Facility type**		
Acute care hospital	Referent	
Outpatient clinic	1.51 (1.00–2.28)	
Nursing home	0.78 (0.52–1.17)	
Other facility type^c^	0.73 (0.57–0.94)	
**Healthcare role**		
Physician	Referent	Referent
Administrative personnel	2.35 (1.49–3.72)	2.25 (1.41–3.57)
Certified nursing assistant	3.49 (2.06–5.92)	2.34 (1.35–4.04)
Medical assistant	2.20 (1.23–3.96)	1.92 (1.06–3.48)
Registered nurse	1.68 (1.15–2.45)	1.40 (0.95–2.06)
Other role anticipated to have substantial patient contact^d^	1.56 (1.04–2.33)	1.37 (0.91–2.07)
Other role anticipated to have moderate patient contact^e^	2.06 (1.33–3.19)	1.90 (1.22–2.95)
Other role anticipated to have minimal patient contact^f^	2.48 (1.46–4.21)	2.34 (1.38–3.99)
Other role with undefined patient contact	0.81 (0.44–1.48)	0.73 (0.40–1.36)
**Close contact with patients with COVID-19 in the workplace in the 14 d before illness onset or SARS-CoV-2 test specimen collection date**		
Yes	1.18 (0.97–1.43)	
No	Referent	
**Assisted COVID-19 patients with activities of daily living in the workplace**		
Yes	1.56 (1.23–1.97)	1.70 (1.31–2.20)
No	Referent	Referent

Note. OR, odds ratio; aOR, adjusted OR; COVID-19, coronavirus disease 2019; SARS-CoV-2, severe acute respiratory syndrome coronavirus 2; SVI, social vulnerability index.

a Highest quartile of SVI values.

b Lowest 3 quartiles of SVI values.

c Includes pharmacies, urgent-care clinics, free-standing emergency rooms or departments, and mental health facilities.

d Includes dental healthcare provider, emergency medical services personnel, licensed practical nurse, nurse practitioner, occupational therapist, other nurse, physician assistant, physical therapist or assistant, phlebotomist, respiratory therapist, radiology technician, speech-language pathologist, and surgical, medical, or emergency technician.

e Includes nonphysician behavioral health provider, chaplain, care coordinator, dietician, environmental services personnel, food services personnel, patient transport personnel, research personnel, social worker, or student.

f Includes facilities maintenance personnel, medical equipment technician, laboratory personnel, or pharmacist.

**Table 4.** Multivariable Logistic Regression Model Estimating Associations Between Social Vulnerability Index (SVI) Themes and SARS-CoV-2 Infection Among Healthcare Personnel, 5 US Emerging Infections Program sites, May–December 2020

SVI theme	aOR (95% CI)^a^
Socioeconomic status	1.77 (1.30–2.39)
Household characteristics	1.36 (1.05–1.78)
Racial and ethnic minority status	0.80 (0.60–1.06)
Housing type and transportation	0.91 (0.71–1.15)

Note. aOR, adjusted odds ratio; CI, confidence interval; COVID-19, coronavirus disease 2019; SARS-CoV-2, severe acute respiratory syndrome coronavirus 2.

a Logistic regression model includes 4 SVI themes as independent covariates as well as healthcare role and assisting COVID-19 patients with activities of daily living in the workplace.

## Discussion

This case–control analysis included 2,168 HCP working in healthcare facilities in 5 US states. We used data from interviews and residential address to characterize census tract-level residential social vulnerability. Compared with controls, cases had 1.8-fold higher odds of residing in highly socially vulnerable census tracts, after controlling for HCP role and assisting COVID-19 cases with their ADL. A more granular analysis of the SVI component themes showed that census tract socioeconomic status and household characteristics were the primary drivers of these disparities, with cases having 1.8 times the odds of residing in tracts ranked most vulnerable with regards to socioeconomic status and 1.4 times the odds of residing in tracts ranked most vulnerable with regards to household characteristics.

Our findings are consistent with numerous population-based studies that found significant associations between high residential social vulnerability and COVID-19 incidence.^21–24^ To our knowledge, this is the first study to characterize residential social vulnerability among HCP and examine its association with SARS-CoV-2 infection while accounting for occupational factors. Taken together with previous data on the significant role of community exposures in transmission,^3,4,7^ as well as racial and ethnic disparities in COVID-19 among HCP,^5–8^ our work contributes to a greater understanding of the social determinants of health that increase risk for SARS-CoV-2 acquisition among HCP. More vulnerable communities may have greater proportions of essential workers who cannot work remotely and workers who live in crowded conditions and multigenerational households, therefore increasing community transmission rates.^21,25^ HCP living in these areas would face additional exposure risk beyond their primary workplaces, especially if they were not able to physically distance while at home or in the community. Additionally, some HCP may work in more than one facility or job. A study of long-term care workers found that a high proportion held a second job or provided unpaid caregiving to someone outside their household, further adding to their exposure risk.^26^

Interestingly, cases were also more likely to be HCP in roles anticipated to have moderate or minimal patient contact, rather than substantial patient contact. This finding may have been due to healthcare facilities prioritizing personal protective equipment (PPE) for those HCP with the most high-risk patient contact during PPE shortages. Staff with substantial patient contact may have received more extensive training on PPE use to prevent infection, both in their formal education and within the workplace. A previous study of PPE use during the first wave of the COVID-19 pandemic found that receiving prior training on proper PPE use, exposure to COVID-19 patients and performing procedures that pose a high risk of exposure to SARS-CoV-2 were significant predictors of PPE compliance.^27^ Importantly, higher proportions of HCP in roles with moderate or minimal patient contact lived in high SVI areas, compared to those with substantial patient contact; therefore, they may have experienced greater risk for community exposures.

Sizeable proportions of Black and Hispanic HCP included in our study lived in high SVI areas. This was an unsurprising finding given that SVI includes a variable for the proportion of the census tract’s population that is a race or ethnicity other than non-Hispanic white. Especially high proportions of CNAs and medical assistants lived in the most vulnerable areas, and cases had higher odds of being in these roles than being physicians.

Much of the guidance to protect HCP from SARS-CoV-2 infection has focused on reducing transmission between patients and HCP, as well as among HCP in occupational settings.^28,29^ Although these are necessary and important interventions, healthcare systems and policymakers should also consider the heterogeneity of social contexts in which the healthcare workforce lives. Healthcare systems could apply measures like the SVI to forecast areas that are especially vulnerable to healthcare worker shortages during future waves of COVID-19 or other pandemics. Attention should also be given to improving the economic conditions of lower-income HCP, as large proportions live in more socially vulnerable areas. For example, a recent study found that raising the minimum wage could reduce the number of female healthcare workers living in poverty by 27%–50%.^30^ Given that Black women are overrepresented in the lower-wage healthcare professions, such policies would further racial health equity in the healthcare workforce.^31^ Providing adequate paid sick leave and health benefits to all HCP, especially those more vulnerable to community exposures, is also necessary to prevent viral transmission to other HCP and patients in the workplace.^32^ Furthermore, given our findings that case status is associated with residing in census tracts with higher proportions of older adults, children, people living with disabilities and single-parent households (ie, the household characteristics SVI theme), healthcare facilities should consider childcare and eldercare policies that can support HCP that are differentially impacted by their household composition and care responsibilities. Finally, focusing interventions to prevent SARS-CoV-2 transmission and social policies to improve economic conditions in the most vulnerable neighborhoods would protect the healthcare workforce as well as the general population.

This study had several limitations. These data were obtained through a convenience sample of healthcare facilities and most participants worked at acute-care hospitals. Testing protocols may have varied among participating facilities. Cases and controls who responded to contact attempts and enrolled in the study may not be representative of all HCP with and without SARS-CoV-2, respectively. HCP were not included in the analysis if they did not provide a complete residential address that could be geocoded to a census tract. Thus, this exclusion criterion may have been applied differentially as more marginalized individuals, such as those with transient addresses, may have been less likely to provide a reliable address. Another limitation is that the exposure source for SARS-CoV-2 cases could not be ascertained, limiting our ability to characterize factors associated with those infected through community versus occupational exposures. Finally, data included in this analysis were collected in the early course of the pandemic, during more widespread implementation of nonpharmaceutical interventions and before availability of COVID-19 vaccines and emergence of the SARS-CoV-2 delta and omicron variants. Although the introduction of vaccines represented a significant advance in the protection of HCP from COVID-19, there is evidence of disparities in vaccine coverage by SVI, and HCP living in high SVI areas are less likely to be vaccinated.^33,34^ Inequities in vaccination may have perpetuated disparities in SARS-CoV-2 infection for HCP living in vulnerable areas. Thus, further work is needed to assess the influence of social vulnerability on SARS-CoV-2 infection in the context of these developments.

In conclusion, in this case–control study, HCP infected with SARS-CoV-2 in 2020 were more likely to reside in more socially vulnerable census tracts compared to controls, after adjusting for HCP role and patient care activities. Residential vulnerability was differentiated by race and ethnicity and healthcare role, with nearly half of Black and Hispanic HCP, as well as CNAs and medical assistants, living in the most vulnerable communities. Our findings also suggest that socioeconomic factors and household characteristics played the most substantial role in driving social vulnerability to SARS-CoV-2 infection in this population. These findings emphasize the need for a holistic understanding of the social determinants of health, both inside and outside the workplace, that increase risk for SARS-CoV-2 infection among HCP. Efforts to improve health equity among HCP should consider the structural forces that entrench racial, ethnic, and socioeconomic disparities in infectious disease outcomes.
